# USE OF OCULOMETRICS AS AN AT HOME BRAIN HEALTH MONITORING TOOL: A CASE STUDY

**DOI:** 10.1093/geroni/igae098.3472

**Published:** 2024-12-31

**Authors:** Quinn Kennedy, Beatriz Hernandez, Dorion Liston

**Affiliations:** neuroFit, Carmel, California, United States; NA, Cupertino, California, United States; neuroFit, Mountain View, California, United States

## Abstract

Quick, objective brain health assessments are needed to monitor long term cognitive function. Oculometrics, the measurement of eye movements, may be one solution as they can quantify the health of cortical circuitry associated with perception and motor tracking. Our previous work demonstrated that an oculometric task is sensitive to a wide range of neurocognitive function. The purpose of this case study was to examine the feasibility of using an oculometric task as an at home brain health monitoring tool. The five-minute task is based on a classic step-ramp visual tracking paradigm that was administered on a PC computer with a camera and eye tracking capability. It yields 10 z-scored eye tracking metrics that are computed into a summary score. A 51-year-old woman completed the oculometric task at home on 138 days over the course of six months and recorded notes regarding anything that may have impacted performance. Data was successfully captured on 111 days (mean score = 0.02 (s =.48)). Distractions in the environment (n = 15 instances) led to worse performance (mean score = -0.37 (s =.51)). Poor sleep the night before (n = 7 instances) also may impair performance (mean score = -.051 (s =.52). Anecdotally, worse scores after poor sleep motivated the participant to improve her sleep habits. Longitudinal plots indicated improvement in the task over the first few weeks, then scores stabilized. Results provide preliminary support for oculometrics as an at-home brain health monitoring tool but point to challenges in controlling environmental factors.

